# Standard method for microCT-based additive manufacturing quality control 1: Porosity analysis

**DOI:** 10.1016/j.mex.2018.09.005

**Published:** 2018-09-15

**Authors:** Anton du Plessis, Philip Sperling, Andre Beerlink, Lerato Tshabalala, Shaik Hoosain, Ntombi Mathe, Stephan G. le Roux

**Affiliations:** a CT Scanner Facility, Stellenbosch University, Stellenbosch, South Africa; b YXLON International GmbH, Hamburg, Germany; c National Laser Centre, Council for Scientific and Industrial Research, South Africa

**Keywords:** Additive manufacturing, MicroCT, X-ray, Tomography, Non-destructive testing, Standardization, Porosity

## Abstract

MicroCT is a well-established technique that is used to analyze the interior of objects non-destructively, and it is especially useful for void or porosity analysis. Besides its widespread use, few standards exist and none for additive manufacturing as yet. This is due to the inherent differences in part design, sizes and geometries, which results in different scan resolutions and qualities. This makes direct comparison between different scans of additively manufactured parts almost impossible. In addition, different image analysis methodologies can produce different results. In this method paper, we present a simplified 10 mm cube-shaped coupon sample as a standard size for detailed analysis of porosity using microCT, and a simplified workflow for obtaining porosity information. The aim is to be able to obtain directly comparable porosity information from different samples from the same AM system and even from different AM systems, and to potentially correlate detailed morphologies of the pores or voids with improper process parameters. The method is applied to two examples of different characteristic types of voids in AM: sub-surface lack of fusion due to improper contour scanning, and tree-like pores growing in the build direction. This standardized method demonstrates the capability for microCT to not only quantify porosity, but also identify void types which can be used to improve AM process optimization.

## Graphical abstract

**Figure:**
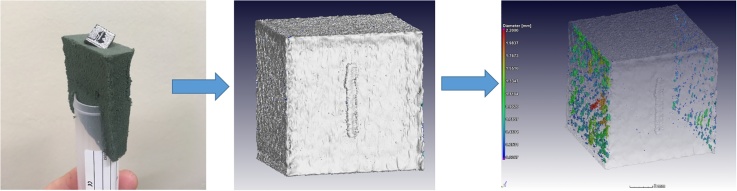

## Introduction

Additive manufacturing (AM) is a fast growing and reliable manufacturing method, with critical metal parts for medical and aerospace applications being produced and processing workflows qualified for the purpose, see for example [1]. Despite huge advances, there are a lack of standards especially for microCT based inspection of AM parts [2]. The microCT-based non-destructive analysis of additive manufactured parts was reviewed recently in [3]. Additive manufactured parts are typically prone to defects such as voids or porosity, which negatively affect their mechanical performance. In the ideal scenario, defects should be minimized in size and extent, which can be achieved through process parameter optimization [4]. Although inspection of the final built part non-destructively is also important, the achievable resolution of X-ray microCT is limited by part size. Typically microCT resolution scales linearly with part size, e.g. 50 mm part results in 50 μm resolution, 20 mm part results in 20 μm, etc. [5] Since some types of additive manufacturing defects may be small and hence missed in a scan of a large complex part, process optimization should ideally be achieved prior to building critical parts. Such process optimization can be done using microCT of small test samples.

Due to the widespread use of microCT for porosity analysis, there are many possible scanning and analysis workflows and algorithms in use for this purpose. Depending on the scan quality (the presence of artefacts, noise, image blur, etc), the type of de-noising used and the analysis workflow used in image processing, different results can be obtained. These can also be presented in different ways which may lead to misinterpretation. Some examples of microCT-based porosity analysis of AM parts are presented in [[6], [7], [8]]. The lack of standards is related to the fact that every type of part tested requires different scan parameters for best quality [9,10]. This has created an opportunity for standardization in the testing of AM parts [2,11], and for microCT of coupon samples. In this work a standard workflow is proposed, with high quality scan parameters, without de-noising and an image analysis workflow with minimal user influence. The coupon sample used is small enough to allow high resolution and quality scans, and besides the porosity analysis demonstrated here, average density and surface roughness can also be quantified on the same sample, as will be demonstrated in subsequent method papers [12,13].

## The method

**Fig. 1:**
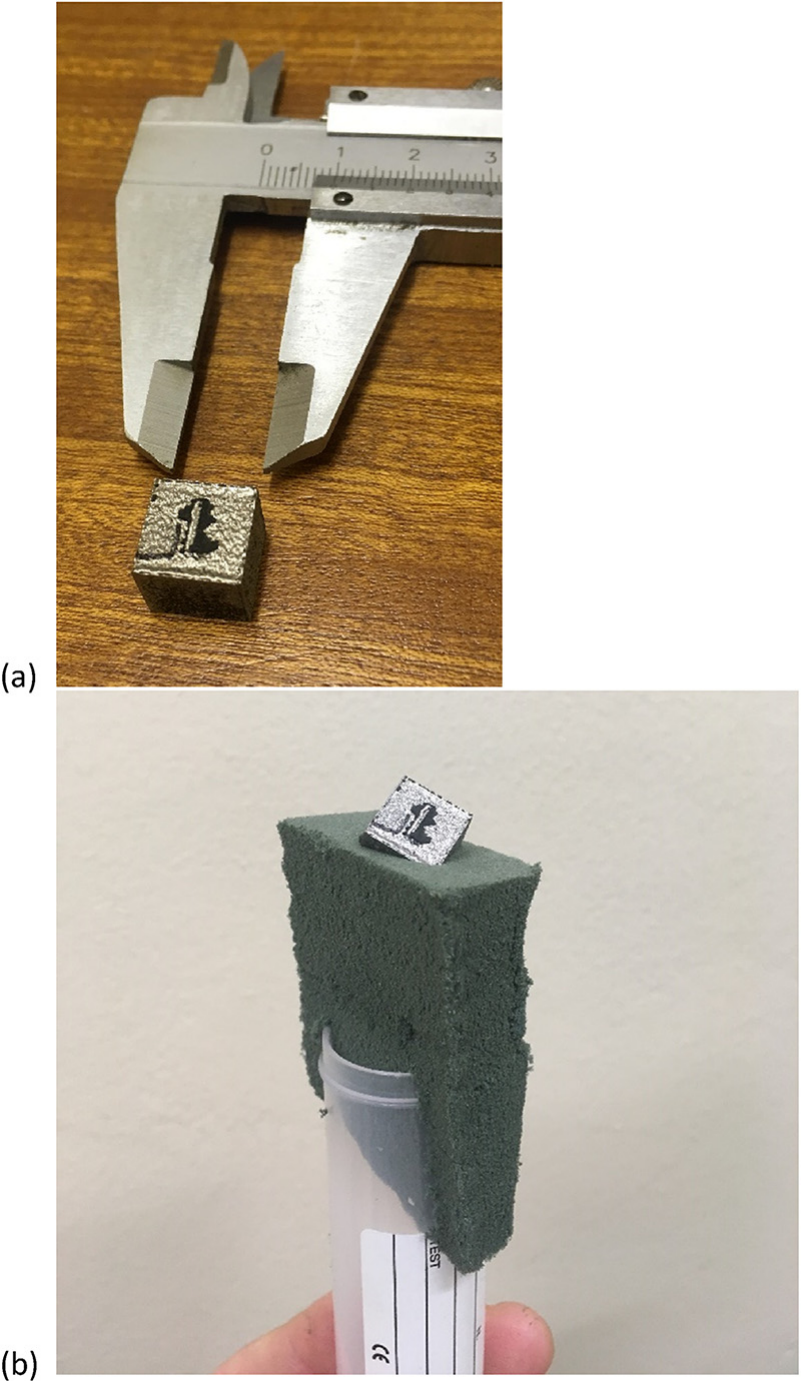
SLM produced Ti-6Al-4V coupon; (a) as-built, (b) sample mounted on foam for a scan.

The samples were built on a custom built selective laser melting platform within a commercial LENS enclosure. The laser used was an IPG YLS 5000 ytterbium 5 kW fibre laser, wavelength of 1076 nm with a delivery fibre core diameter of 50 μm. The scanner used was an Intelliweld 30 FC V system. Materials used were Ti6Al4V provided by TLS Technik GmbH, gas atomized with particle size 20–60 μm. The base plate material is Ti6Al4V 150 mm in diameter approximately 40 mm thick. The hatch parameters used were a power of 3 kW, speed of 3 m/second, and a 240 μm spot size. The contour scans used a laser power of 1 kW where the distance between the hatch and contour and the speed was varied to investigate its effect on density and surface finish. A standard coupon sample of 10 × 10 × 10 mm is used for this test as shown in Fig. 1a. This test is for the widely used aerospace and medical Ti6Al4V alloy, but may be applied identically to other light metals and plastics. For steels or heavier metals, a higher voltage is required in order to provide sufficient penetration, and stronger beam hardening correction must be applied in the reconstruction step.

This sample size allows for a high enough scan resolution to image all types of additive manufacturing defects (15 μm), while allowing a large enough sample size for practical purposes (and field of view size). All scanning and image analysis steps are described for standardizing the method, and importantly, there is very limited human selection in the process and most bias is therefore removed. This method can be cost effective considering the additional information obtained visually regarding the root cause of density variations such as porosity or unconsolidated powder.

The sample is loaded in foam at an angle of roughly 10–45°: this ensures that no edge artefacts are present, as shown in Fig. 1. MicroCT is performed using a standard laboratory microCT system [9], with parameters optimized according to the guidelines presented in [10]. MicroCT scan settings of 200 kV, 70 uA, with 0.5 mm beam filter are used, with image acquisition of 500 ms per image, 2400 step positions in a full 360 ° rotation. At each step position, the first image is discarded and two subsequent images averaged. The total scan time is approximately 1 h. When sample setup, machine warmup, background correction and reconstruction is included this should be possible in almost any system in 2 h total. The reconstruction is done using a strong beam hardening correction factor without any image de-noising.

The data is then analysed in Volume Graphics VGStudioMax 3.1 software. The image processing steps are described here in detail for removing the exterior air from the data set, but including all material and air (open and closed pores, not surface roughness). This may be done in different methods and with different softwares, the aim is to create an accurate segmentation of the edge of the cube irrespective of the size of internal pores or possible external noise particles (eg in the foam), in this case in an automated method is presented. A supplementary video demonstrates the simplicity of the process despite the seemingly complex description.

This segmentation is achieved by firstly applying a basic “automatic” surface determination, followed by creating a region of interest (ROI) from this surface. This region is then modified by an opening/closing function with a value of +3 and creating a new ROI, which closes up small surface pores resulting from the surface roughness. A region growing tool is then used with high tolerance (no effect of grey values) on the air surrounding the part, while the option is selected for “avoid other visible ROIs”. When performing this step, make sure that the “volume” is highlighted and the ROI is visible. This selects all exterior air up to the edge of the part as designated by the surface determination and surface closing function. If small noise particles (for example loose powder) are present outside the part, an opening/closing function (+3) can be applied to this region, to remove these from the selection. Inverting this exterior-air selection allows to select the entire part including its internal voids. A new advanced surface determination function is then applied, using this ROI selection as a starting contour. In this way the local optimization is performed on the exterior surface, allowing the best sub-voxel precision on the surface location.

**Fig. 2:**
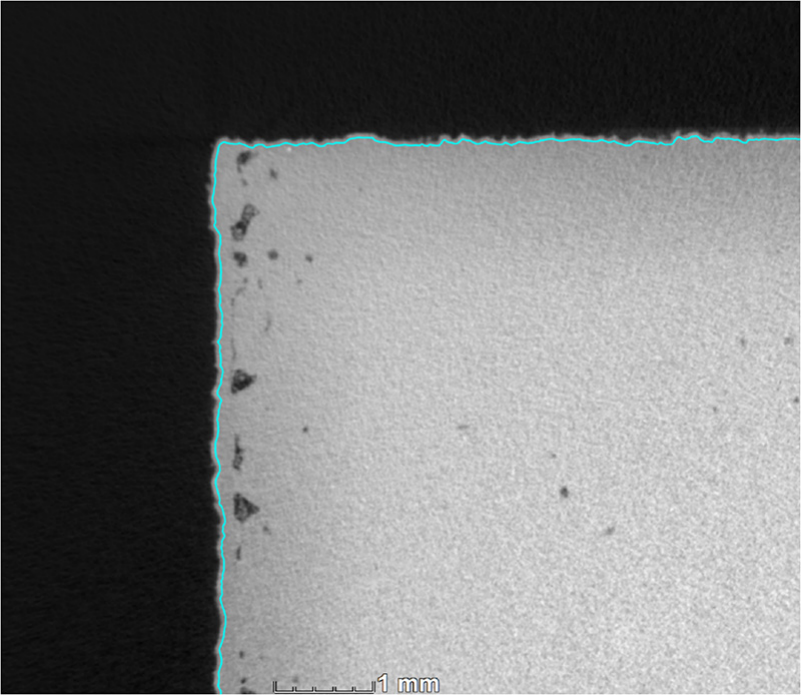
Segmentation of subsurface region to remove edge errors on the part. Deeper subsurface porosity is also shown.

The quantitative defect (porosity / void) analysis can be done in many ways, but a direct segmentation method is described here which does not use any special algorithm and is therefore less susceptible to errors in (possibly) noisy data. Since the as-built parts have a rough surface, and to eliminate other edge errors, we propose here to select a region of interest sub-surface by 2 voxels, using the erode function (erosion −2). First use the “select ROI from surface” to create an ROI, go to selection modes “erode/dilate” and erode the sample by −2, create a new volume and extract the ROI. This eliminates (open) edge pores on the rough surface (in a skin distance of approximately 30 μm). Fig. 2 shows this selection with a blue line, on one corner of the cube.

After extracting this region, a new advanced surface determination is applied to the material/defect interface regions, using the new ROI as described in the previous section. This interface threshold must be selected manually in a properly contrasted slice view with the preview function, to ensure the threshold between pore space and material is properly selected (see video in supplementary material). The local optimization then performs a refinement of this selection, assisting to correct any possible human bias in this selection. If no voids are visible in any slice image, the threshold must be selected to the left of the material peak which delivers a zero result. The selected region is inverted to highlight only void spaces. The inverted region (therefore including all voids / pores) is used as a defect mask in the porosity analysis function.

**Fig. 3:**
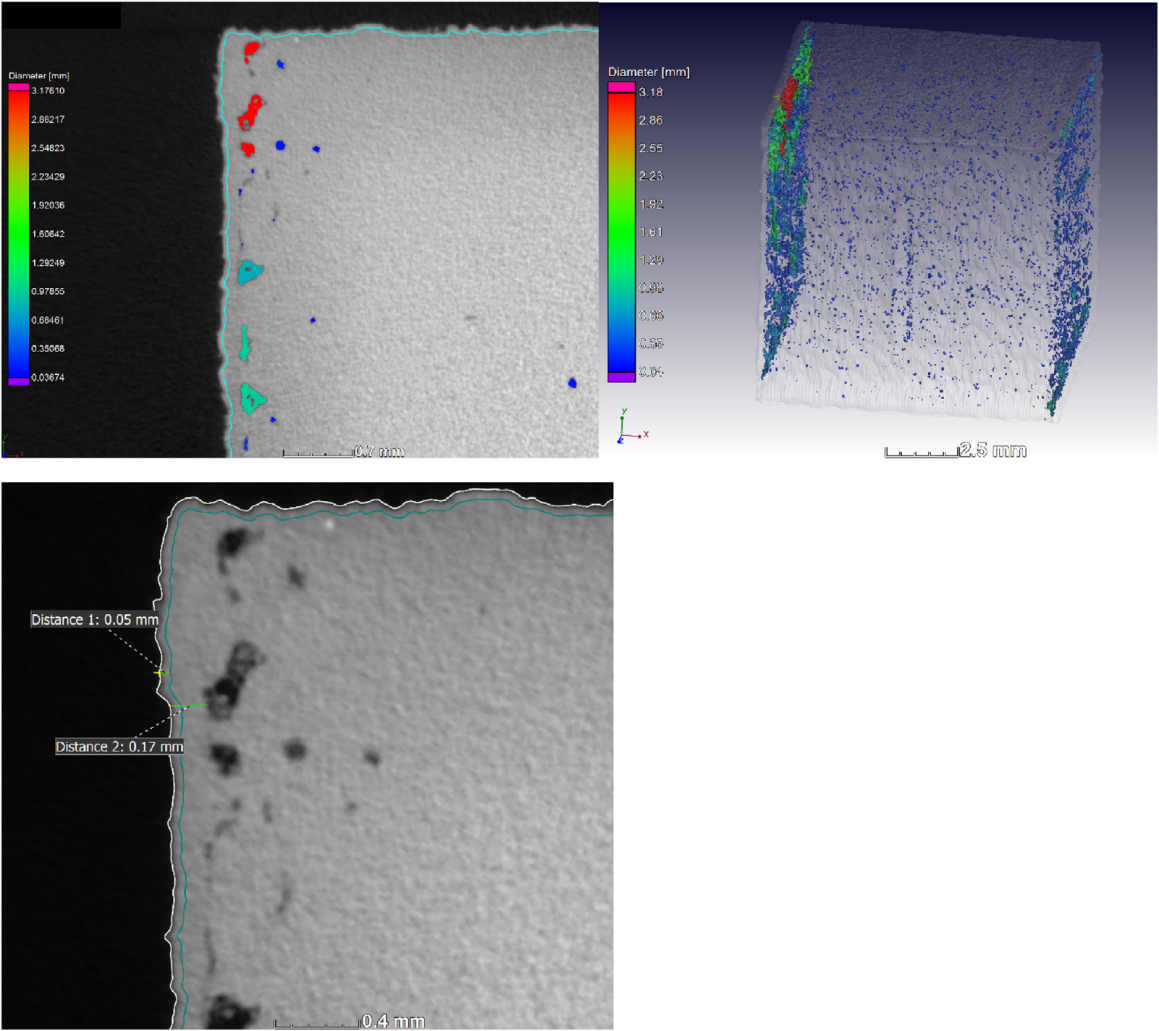
Defect analysis of a coupon sample – the total porosity is 0.24% and located near the surface indicating the effects of improperly selected contouring tracks: (a) shows the slice view with segmentation line in blue, and porosity with colour coding, (b) shows the 3D view of the porosity and (c) shows the depth of the segmentation line (∼50 μm) and the subsurface porosity (∼170 μm) relative to surface (white line).

Fig. 3 shows the result for a sample with mainly near-surface porosity. The diameter given is the circumscribed sphere, in other words the longest cross section of the pore in each case. The subsurface porosity shows that a contour scanning error is present in the AM system used in this case.

**Fig. 4:**
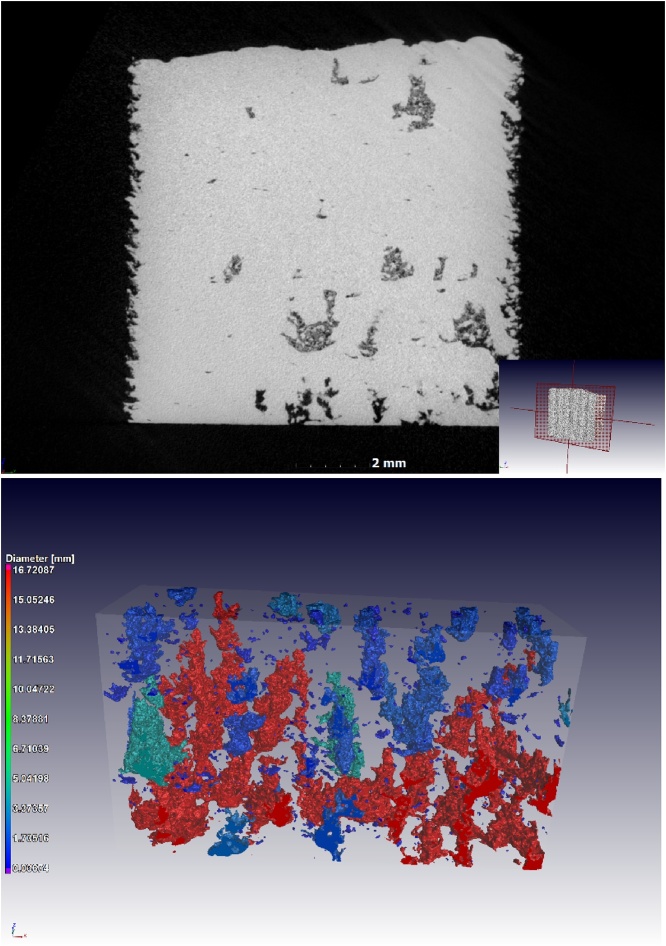
Images of tree-like voids grown in the build direction, with unconsolidated powder trapped inside the closed voids: (a) slice view and (b) 3D rendering.

Another type of defect is shown in Fig. 4 - tree-like porosity growing in the build direction, containing unconsolidated powder. Some of these voids are open to the surface, which allowed the powder to exit, resulting in darker void spaces. The segmentation method can be applied to either include or exclude the powder-filled voids, in this example they are included.

The workflow described in this paper is not entirely independent of human error, since the threshold step requires human intervention. In order to automate the process, algorithms are available for automatically assessing the local greyvalue variations to assign pore or void spaces. However, the manual method eliminates possible errors such as in the case of powder filled voids which might be missed by automated algorithms. It is also possible that small pore spaces can be seen but not included in the analysis, due to partial volume effects. This is acceptable as the smallest pores in the segmentation are selected to be 8 voxels (2 × 2 × 2), in order to eliminate noise. In the case of such interest in further detailed analysis, sectioning and higher resolution scans or microscope-based imaging is suggested. The second in this series of papers discusses mean density determination, which can also overcome this problem to some extent, especially when large numbers of pores are smaller than the voxel size [12].

**Fig. 5:**
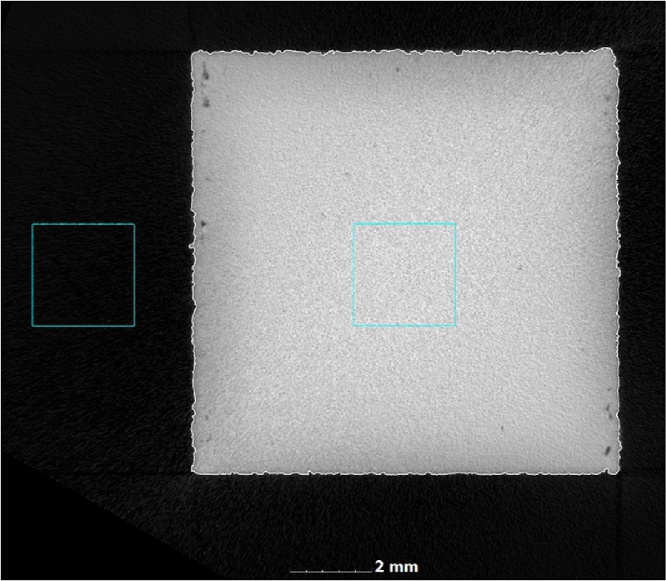
Measuring image quality: cubic volumes selected inside and outside the part.

**Fig. 6:**
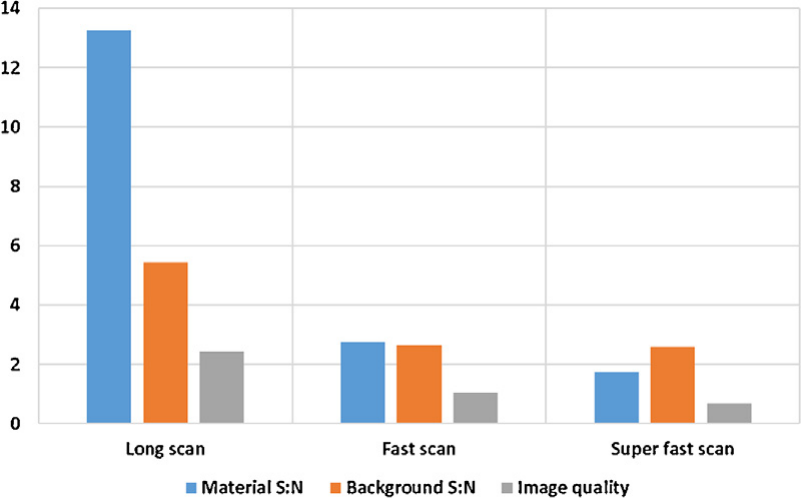
Image quality for optimized scan of 1 h, faster scan of 25 min and fast scan of 1 min.

In order to make a quantitative analysis of voids or porosity, the CT scan image quality is crucial, which is mainly determined by the scan time. We demonstrate here a simple method to measure the image quality in CT images: a 2.5 mm cube is selected inside the coupon sample 10 mm cube and provides an average grey value and a standard deviation of grey values within this cube. The ratio of grey value over standard deviation of grey values provides a signal to noise ratio for the material. Similarly a signal to noise ratio for the background is obtained. The cubes selected is shown in Fig. 5. The ratio of signal to noise for material over background provides an overall image quality metric that can be applied to any CT data sets. It is important to realize that any image processing will affect this result – for example applying a de-noising filter during reconstruction will reduce the variation of grey values thereby increasing the signal to noise. Therefore this criteria must be used on unprocessed data, and potentially additional image quality metrics need to be devised when comparing different systems, this is currently under investigation. Here we propose it only as a simple system for generic indication of good image quality when comparing different scans on the same system. In this case we demonstrate the metric for three types of scans in Fig. 6, the 1 h scan being the one used here for quantitative analysis. As the image quality reduces, the images become grainy and more noisy in material and in background, making it more difficult to accurately detect pores, and easier to make mistakes (false pore identifications).

**Fig. 7:**
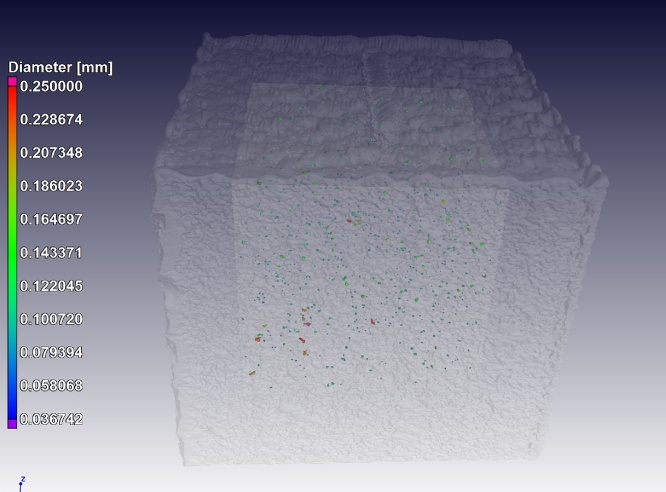
Porosity analysis of inner 7 mm cube of sample – 0.023% average porosity, largest pore size 266 μm.

**Fig. 8:**
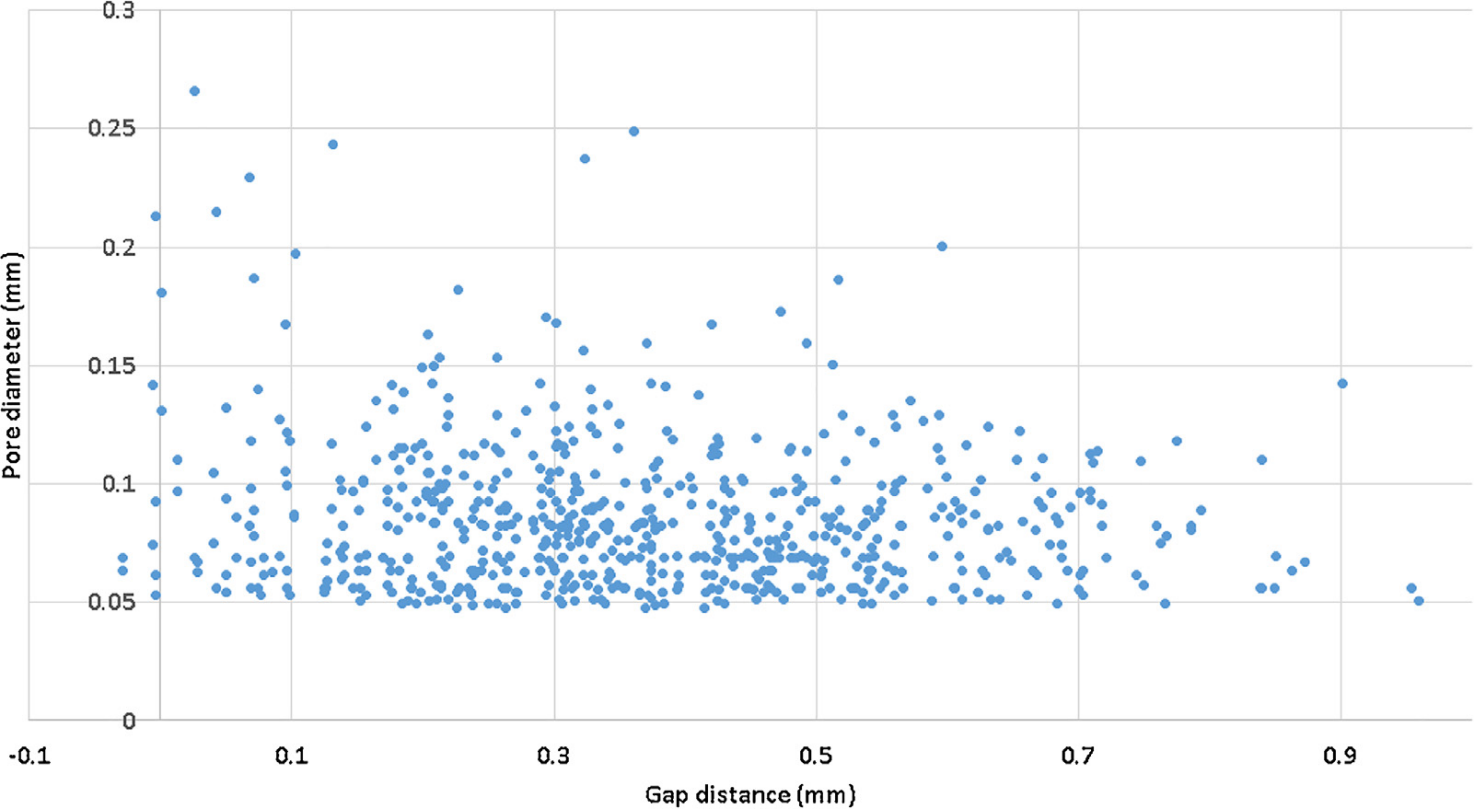
Pore diameter vs gap distance for pores from inner part of sample (7 × 7 × 7 mm cube) – this can be used to assess the proximity of pores relative to one another and make pass/fail decisions.

Besides total porosity, the largest pore and pore distribution, another specification in aerospace applications is the distance between pores relative to their size. Specifically, the distance between pores must be larger than the diameter of the largest pore of the two under consideration. This can be automatically analysed using the gap distance – which provides for each pore the distance to the next nearest pore space (minimal distance between circumscribed spheres of the pore spaces). This is demonstrated on a subvolume of 7 mm in the same sample shown in Fig. 1, this time excluding the near-surface pores. Fig. 7 shows the presence of a total of 648 pores with an average volumetric porosity of 0.023%, and Fig. 8 shows the gap distance vs the pore diameter. A simple calculation shows that in this case 42 pores fail the criteria for proximity to other pores.

The two types of flaws demonstrated in this paper were retrospectively identified as due to contour scanning effects for the subsurface pores – in this case the filling scan tracks and the exterior contour scan tracks where slightly too far apart, creating voids between them along the vertical walls of the part. In the case of the treelike pores, this occurred due to serious lack of fusion, causing the start of an area of improper powder spreading, which results in subsequent layers having increasing amounts of porosity all connected to the layers underneath. This lack of fusion was due to very fast scanning and hence lack of sufficient energy input per layer, making the likelihood for this type of porosity increase with scan speed. These type of flaws can therefore also be seeded into test samples but further work in this direction needs to be done to produce flaws of well known extent, possibly combined with microCT according to the methodology outlined in this paper. Some previous work has included the seeding of cavities in test samples as shown in [6] and in [8].

## Conclusion

We have demonstrated a dedicated and simple workflow for direct void / porosity analysis in 10 mm coupon samples produced by AM. This workflow requires minimal user choice, assisting in standardization. Further automation of the workflow is possible when using image de-noising and automated porosity analysis algorithms, but this might depend on individual analysis requirements. The workflow presented here is simple and can be verified during the image analysis process. We envisage this method to be useful for process parameter optimization and detailed analysis of pore types, identifying AM processing errors.

## Appendix A Supplementary data

A video is included which demonstrates the image analysis workflow step by step. The analysed volume data for one sample is available and will be published online and made freely accessible.
